# Seizure: An Adverse Effect of Regadenoson in Myocardial Perfusion Imaging

**DOI:** 10.1155/2019/6240605

**Published:** 2019-04-08

**Authors:** Sohab S. Radwan, Owen G. Schwartz

**Affiliations:** Department of Internal Medicine, MedStar Washington Hospital Center, USA

## Abstract

Regadenoson is a selective adenosine 2a (A2a) receptor agonist that is used in cardiac stress testing to evaluate for ischemic heart disease and has largely replaced adenosine in the modern era. Since adenosine receptors are involved in synaptic transmission between neurons throughout the central nervous system (CNS) including the cerebral cortex, hippocampus, and other structures as well, regadenoson can lower the seizure threshold in susceptible individuals. Epileptogenic activity is an uncommon yet potentially severe adverse effect of regadenoson use, and therefore, more awareness is required in screening patients at risk and evaluating alternate ways to investigate coronary artery disease (CAD) in susceptible individuals.

## 1. Introduction

Regadenoson is a selective A2a receptor agonist that acts on coronary artery smooth muscle to promote vasodilatation, increasing myocardial blood flow. It is widely used in rest-pharmacological stress myocardial perfusion imaging (MPI) as part of cardiac testing to evaluate for CAD, especially in patients with limited exercise capacity. It has largely replaced adenosine in MPI due to a better safety profile. However, adenosine receptors and their subtypes are present in several extracardiac tissues resulting in cross reactivity and potential toxicity. One example is that adenosine receptors play a role in neuroregulation and excitatory glutamatergic transmission; thus, regadenoson can lower seizure threshold through A2a receptor stimulation. We hereby present a case of seizure provoked by regadenoson administration and subsequently discuss the underlying mechanism.

## 2. Case Presentation

A 74-year-old female with a past medical history of hypertension, diabetes mellitus type 2, CAD status post coronary artery bypass grafting, and ischemic stroke with residual left-sided weakness presented to the emergency department with a one-day history of retrosternal chest pain radiating to her left shoulder. On presentation she was normotensive, and electrocardiogram (EKG) revealed sinus rhythm with a heart rate of 72 beats per minute, left axis deviation, and T wave inversion in leads I and aVL which were unchanged from a prior EKG several months ago. Initial troponin I was negative. Based on her risk factor profile and pretest probability for CAD, she was scheduled for a rest-pharmacological stress MPI test. While at rest, the patient was injected intravenously with 99mTc-tetrofosmin and images were acquired approximately 45-60 minutes later with 180-degree single-photon emission computerized tomography (SPECT). Subsequently, the patient was injected with 0.4 mg of regadenoson over 15-20 seconds while being monitored by12-lead EKG. Approximately 30 seconds after regadenoson injection, the patient was injected with 99mTc-tetrofosmin and 180-degree SPECT images were taken approximately 45 minutes later. The gated SPECT images revealed normal rest and stress tetrofosmin perfusion, as well as a normal left ventricular function. Approximately 120 minutes after regadenoson administration, the patient developed a generalized tonic-clonic seizure that lasted for 3 minutes. On initial assessment, she was hemodynamically stable and not hypoxic. She received 2 mg of lorazepam and 1 g of levetiracetam intravenously, which aborted the seizure. She did not receive aminophylline. On physical examination after she regained consciousness, she was confused and not oriented. Cranial nerves were intact, and motor strength was unchanged from baseline (5/5 at the right upper and lower extremities and 4/5 at the left upper and lower extremities). Initial blood work revealed sodium of 142 mmol/L, potassium 3.8 mmol/L, glucose 5.82 mmol/L, calcium 2.17 mmol/L, and magnesium 0.78 mmol/L. Due to concern for a new cerebrovascular accident in setting of prior history and this acute event, she underwent an emergent brain magnetic resonance imaging (MRI) study which did not reveal any acute changes. She was started on levetiracetam 500 mg orally twice daily. An electroencephalogram (EEG) was performed and revealed no epileptiform abnormalities or epileptogenic foci. Of note, she was not taking any antidepressants such as bupropion or other medications that may lower seizure threshold. She was discharged home 4 days later, with a diagnosis of regadenoson-induced seizure.

## 3. Discussion

Regadenoson is a selective A2a receptor agonist, which is predominant in coronary artery smooth muscle. However, it is also a weak agonist at A1a receptors with negligible affinity to A2b and A3a receptors as well [1]. It is commonly used as a pharmacological stress test agent to assess coronary blood flow in patients with limited functional capacity who cannot undergo exercise stress testing. When compared to adenosine, regadenoson was associated with a greater and more rapid peak in heart rate and demonstrated noninferiority in detecting perfusion defects with a better safety profile [2, 3]. However, given the wide range of adenosine receptors and their subtypes, a diverse adverse effect profile of regadenoson has been reported including nausea, vomiting, dyspnea, and headache [4]. Other side effects such as atrioventricular block, bronchoconstriction, and flushing have been attributed to its agonistic effects on A1a, A2b, and A3a receptors, respectively, despite low affinity [1, 5].

Seizure is one of the recently recognized adverse effects of regadenoson with increasing incidence. As of June 2018, a total of 148 cases of seizures and 10 cases of seizure-like phenomena were reported to the US Food and Drug Administration as adverse effects of regadenoson. Of note, the population size exposed to regadenoson, as well as the average time from injection to seizure onset, and past history of seizures or strokes in those patients have not been reported to the FDA [4]. A2a receptors are widely distributed in the CNS including the cerebral cortex, striatum, nucleus accumbens, and hippocampus [6]. Adenosine, specifically, through A2a receptors plays a role in glutamate outflow tracts and thus is involved in excitatory neurotransmission between neurons [7]. Several animal models, specifically rodents, have been studied regarding the role of A2a receptors in neuronal interaction, as well as the clinical application of adenosine receptor antagonists in treatment of seizures. One study investigated the consequences of deleting the A2a receptor in mice by comparing the incidence of electroshock-induced seizures in A2a-knockout mice versus controls [8]. Results showed that A2a-knockout mice demonstrated lower percentage as well as intensity of seizures than controls [8]. In another study, bilateral striata of rats were lesioned using the excitotoxin quinolinic acid, and this was followed by the administration of SCH 58261 (potent A2a receptor antagonist) [7]. Those who received SCH 58261 showed significantly lower motor, electroencephalographic, and neuropathologic changes secondary to excitotoxin [7]. Despite such findings, the clinical application of adenosine receptor antagonists in the treatment of epilepsy is still under investigation.

Regarding the pharmacokinetics of regadenoson, the elimination half-life follows a 3-compartment model [9]. The half-life of the initial phase is approximately 1-4 minutes, followed by an intermediate phase which has a half-life up to 30 minutes [9]. The final and terminal phase has a half-life lasting up to 2 hours [9, 10]. Most adverse effects begin soon after regadenoson administration and resolve usually within 15 minutes; though of those reported, the majority involves bronchospasm and cardiac conduction blocks [11]. Moreover, a case series by Page et al. demonstrated 3 cases of regadenoson-induced seizures that developed within 2-5 minutes of administration [12]. Two cases had a prior medical history of seizures and were receiving antiepileptic drugs, while one had no prior seizure history which however displayed hemodynamic instability prior to seizure onset [12]. On the other hand, it has been reported that seizure onset following regadenoson administration may be delayed as well, but no specific time onset was defined [13, 14]. For such reasons, aminophylline, a nonselective adenosine receptor antagonist, has been used to prevent delayed adverse effects, especially in patients at higher risk, such as a prior history of seizures [14]. Although it potentially antagonizes adenosine receptors, aminophylline is not a good option for reversal of regadenoson-induced seizures, as it may be sometimes ineffective in such setting and may even lower seizure threshold further and thus provoke seizures [12].

While the exact incidence of regadenoson-induced seizures is unknown, more cases are being reported as discussed above. We believe that the seizure in our patient may have been provoked by regadenoson, since other organic and structural causes were ruled out. The American Society of Nuclear Cardiology (ASNC) recommends avoiding regadenoson in patients with a medical history of seizures and structural brain disease and lists it as a relative contraindication to this medication [15]. We believe that our case adds to the available literature describing this uncommon but potentially severe adverse effect of regadenoson. It also advocates for increased physician awareness in screening patients at risk and providing prompt detection and appropriate management. It is also imperative to remember that aminophylline should be used with caution in reversal of regadenoson-induced seizure as indicated above. Additionally, nuclear medicine physicians and technicians need to be aware of such adverse events, and appropriate acute seizure termination measures should be always easily accessible.
